# The effects of immunotherapy with intravenous immunoglobulins versus no intervention, placebo, or usual care in patients with recurrent miscarriages: a protocol for a systematic review with meta-analyses, trial sequential analyses, and individual patient data meta-analyses of randomised clinical trials

**DOI:** 10.1186/2046-4053-3-89

**Published:** 2014-08-15

**Authors:** Pia Egerup, Jane Lindschou, Christian Gluud, Ole Bjarne Christiansen

**Affiliations:** 1 The Fertility Department, Rigshospitalet, Copenhagen University Hospital, Copenhagen, Denmark; 2 Copenhagen Trial Unit, Centre for Clinical Intervention Research, Rigshospitalet, Copenhagen University Hospital, Copenhagen, Denmark

**Keywords:** Systematic review, Meta-analysis, Recurrent miscarriage, Immunotherapy, Immunoglobulins

## Abstract

**Background:**

Recurrent miscarriage is generally defined as three or more miscarriages before gestational week 20. Recurrent miscarriage affects 1% of all women and the condition can only be explained by parental chromosome abnormalities, uterine malformations, or endocrine or thrombophilic disturbances to a limited extent. Immunological disturbances are hypothesised to play an important role in recurrent miscarriage and, therefore, various types of immunologically-based therapies have been tested in recurrent miscarriage patients including intravenous immunoglobulins. So far, at least eight randomised placebo-controlled trials, with opposing results, investigating intravenous immunoglobulins with a total of 324 recurrent miscarriage patients have been published.

**Methods/Design:**

We will include randomised clinical trials irrespective of publication date, publication type, publication language, and publication status investigating infusions with immunoglobulins in relation to pregnancy compared to placebo, no intervention, or treatment as usual for assessments of benefits and harms. The relevant published literature will be searched using the following databases: Cochrane Central Register of Controlled Trials, Medline, Embase, WHO International Clinical Trials Registry Platform, and Ovid Medline In-Process and Other Non-Indexed Citations databases. Two review authors will independently extract data and assess risk of bias. We will undertake meta-analyses according to the recommendations stated in the *Cochrane Handbook for Systematic Reviews of Interventions*. Further, we will conduct trial sequential analyses and individual patient data meta-analyses.

**Discussion:**

A miscarriage results in great sorrow, loss of life quality, and personal concern. In particular, recurrent miscarriage is extremely stressful and burdensome. It is, therefore, very important to conduct research in this area. There is currently no evidence-based treatment for women with recurrent miscarriage which significantly improves their ability to give live birth. Therefore, a comprehensive up-to-date systematic review is needed. By using individual patient data, it will be possible to provide new knowledge about the benefits and harms of intravenous immunoglobulins and try to identify the subgroup in which the treatment will have the highest impact.

This systematic review protocol was registered within the International Prospective Register of Systematic Reviews (PROSPERO) as number CRD42014007112.

## Background

Recurrent miscarriage (RM) is generally defined as three or more miscarriages before gestational week 20 [1]. However, many clinicians define RM as two or more miscarriages [2]. Primary RM refers to a series of miscarriages without a previous live birth. Secondary RM refers to a woman with a series of miscarriages subsequent to a previous live birth [1]. In some clinics it is called secondary RM if the miscarriage has been preceded by a live birth or stillbirth after gestational week 22 [3]. RM affects 1% of all women and only in a minority can the condition be explained by parental chromosome abnormalities, uterine malformations, or endocrine or thrombophilic disturbances [1].

Immunological disturbances are hypothesised to play an important role in RM. Elevated levels of natural killer cell subset, autoantibodies, and inflammatory cytokines can be found in the peripheral blood of these patients and significantly more activated leukocytes and abnormal levels of specific natural killer cell subsets in the decidua of women with RM have also been described [4-7]. There is some evidence that immunological disturbances play a larger role in secondary RM compared to primary RM. There is a higher prevalence of the immunological high responder HLA allele HLA-DR3 and specific HLA-G genotypes in secondary RM than in primary RM and controls without RM [7,8]. A study also shows that there is an excess of boys born prior to secondary RM and an excess of live born girls in women giving birth after secondary RM compared to the expected 1:1 sex ratio [9]. This gives rise to the hypothesis that women with RM have developed a harmful immunological reaction against male-specific minor histocompatibility antigens (HY-antigens) on the foetus or trophoblast, and that this results in subsequent increased miscarriage rate of male conceptions [9].

Because of the association between these immunological biomarkers and RM, various types of immunologically-based therapies have been tested in RM patients. Until now, four different kinds of immunotherapy have been tested in placebo-controlled trials: prednisone, immunisation with trophoblast membrane, active immunisation with allogeneic lymphocytes from the partner or donors, and intravenous immunoglobulins (IvIg) [10-12].

IvIg have several effects like suppression and neutralisation of autoantibodies, attenuation of natural killer cells, inhibition of complement binding, modification of cytokine production, and expansion of regulatory T lymphocytes [13-15]. IvIg exhibit a documented effect in many disorders caused by immunological abnormalities [16]. IvIg are made by extracting the IgG fractions from plasma from normal blood donors and, therefore, there are potential risks of adverse events like allergy and transmission of infections (for example, HIV, hepatitis, prions). In general, IvIg are well tolerated, and the most frequent adverse reactions, which include headache, fever and nausea, occur in less than 5% of patients [13].

So far, at least eight randomised placebo-controlled trials investigating IvIg with a total of 324 RM patients have been published, with conflicting results [12,17-23]. Among these, one found a statistically significant beneficial effect [17], one found a strong trend towards a beneficial effect [18] and six showed no effects [12,19-23].

These differences can in theory be explained by the fact that the trials conducted so far are very heterogeneous with regard to the selection of patients, doses of IvIg, and starting time of infusions. The doses used in the trials range from small doses [17,20,22] to doses similar to those used in the treatment of autoimmune diseases [12]. There are also large variations in the starting time for the first infusion and the number of infusions administered in the trials. The patient population differs much in the different trials, especially by numbers of miscarriages and the participation of primary or secondary RM patients. This heterogeneity may blur a possible treatment effect.

There is, in our view, an urgent need for an up-dated systematic review with meta-analysis and trial sequential analyses of randomised trials of IvIg in RM. One reason is that there is a need to conduct important subgroup analyses to evaluate in which subgroup the treatment may be most beneficial. We will, in our systematic review, include data from the newest randomised placebo-controlled trial, Trial of the Efficacy of Intravenous Immunoglobulin for Treating Women With Unexplained Secondary Recurrent Miscarriage (ClinicalTrials.gov Identifier: NCT00722475), which is expected to be published in 2014.

## Methods

### Types of participants

Women who meet the following inclusion criteria will be included:

1) A history of two or more consecutive miscarriages, defined according to the trialists

2) Normal anatomy of the uterine cavity assessed by, for example, hysterosalpingogram, sonohysterogram, vaginal sonography, or hysteroscopy

3) Normal parental karyotypes

4) Pregnancy diagnosed by positive beta-human chorionic gonadotropin (β-hCG) test in serum, or urine, or by ultrasound

5) Informed consent for participation in the randomised trial

### Types of interventions

#### Experimental groups

IvIg initiated before pregnancy or during the first trimester of pregnancy regardless of dose or length of intervention period.

#### Control groups

Placebo: any placebo substance containing no active substance.

No intervention.

Treatment as usual: usual treatment or care, as defined by the trialists.

#### Co-interventions

We will allow all types of co-interventions as long as they are given equally in both intervention groups.

### Types of studies

We will include randomised clinical trials irrespective of publication date, publication type, publication language, and publication status investigating infusions with IvIg in relation to pregnancy compared with placebo, no intervention, or treatment as usual for assessments of benefits and harms. For assessments of harms we will also include the quasi-randomised clinical studies and observational studies that we happen to identify in our search for randomised clinical trials.

We will use individual patient data (IPD) combined with aggregate data (AD) in the data analyses in order to detect a subgroup of patients with most beneficial effect.

### Outcomes

#### Primary outcomes

The primary outcomes are:

1) The proportion of women not giving live birth, defined according to the trialists

2) The proportion of women experiencing a serious adverse event (SAE) defined as any adverse event that results in death, is life-threatening, requires hospitalisation or prolongation of existing hospitalisation, or results in persistent or significant disability or incapacity [24]. SAE will be assessed as a composite of all the above events

3) The proportion of live-born babies experiencing a SAE defined as any adverse event that results in death, is life-threatening, requires hospitalisation in a neonatal care unit or prolongation of existing hospitalisation, results in persistent or significant disability or incapacity or is a congenital anomaly or birth defect [24]. SAE will be assessed as a composite of all the above events

#### Secondary outcomes

The secondary outcomes for all women are:

1) The proportion of women experiencing an adverse event (AE) defined as any undesirable medical event occurring to a participant during a clinical trial, which does not necessarily have a causal relationship with the intervention [24]. AE will be assessed as a composite of all the above events

2) The women’s quality of life, as defined by the trialists

The secondary outcomes for women with a live birth and their babies are:

1) Proportion of women who gives live birth prematurely (<37 weeks)

2) Sex of the baby

3) The proportion of babies with low birth weight, that is < 2,500 g

4) The proportion of babies experiencing an AE. AE will be assessed as a composite

5) The babies’ quality of life, as defined by the trialists

### Searches

The relevant published literature will be searched using the following databases: the Cochrane Central Register of Controlled Trials (Central 2014), Medline (1950 to April 2014), Embase (1947 to April 2014), WHO International Clinical Trials Registry Platform (April 2014), and Ovid Medline In-Process and Other Non-Indexed Citations databases (April 2014). The following medical subject headings (MeSH) terms, keywords and their combinations will be used: immunoglobulins; intravenous; immunotherapy; foetal death, abortion; habitual abortion; spontaneous; foetal loss; miscarriage; recurrent abortion; recurrent miscarriage. Appropriate suffixes will be used for each database. The Cochrane strategy for identifying randomised trials using the relevant MeSH terms and keywords will be used. Similar search strategies will be used in Central, Embase and Ovid Medline In-Process and other non-indexed citation databases. Relevant abstracts from the annual meetings of American and European Societies of Reproductive Medicine and Human Reproduction will be searched. The reference lists of the identified reports will be manually searched for other relevant publications.

No language restrictions will be applied.

### Selection of trials

Two authors (PE and JL) will independently identify trials for inclusion. Firstly, titles and abstracts of the records retrieved by the search will be assessed in order to exclude those that are irrelevant. For the remaining records, full-text articles will be retrieved in order to select trials that meet the inclusion criteria. We will list the trials excluded from the second round and give the reasons for their exclusion. Differences of opinion will be resolved by discussion with a third author (CG).

### Data extraction

Two authors (PE and JL) will independently extract data from all included trials. Any disagreement will be discussed, a third author (CG) will help clarify issues, and these final decisions will be documented. Data presented only in graphs and figures will be extracted whenever possible, but will only be included if two reviewers independently had the same result. Authors of trials may be contacted for clarification.

The following data will be extracted from each of the eligible included trials:

1) Demographic data (country, trial period, number of women randomised, age, exact number of previous miscarriages, primary or secondary RM, levels of IgG anticardiolipin and positivity/negativity for lupus anticoagulant)

2) Design and risk of bias (allocation sequence, allocation concealment, blinding of participants and treatment providers, blinding of outcome assessors, incomplete outcome data, selective outcome reporting, for-profit bias, other sources of bias)

3) Procedural (brand of IvIg, dose and number of infusions, time of first infusion, type of control intervention, co-interventions), and

4) Outcome data (see Outcome section)

When relevant data are not provided in the eligible publications, attempts will be made to obtain them by contacting the original authors.

The original authors will also be contacted with the aim of collecting individual patient data (IPD). The original authors will be invited to become part of the ImmuReM (Intravenous *Immu*noglobulins in *Re*current *M*iscarriage) IPD Study Group, which will co-author the systematic review publication.

We will aim to obtain the following IPD:

Participant-level information before trial entry:

1) Unique identification coded for anonymity

2) Maternal age at inclusion

3) Exact number of previous miscarriages as defined by the authors

4) Primary or secondary RM as defined by the authors

5) Levels of IgG anticardiolipin and positivity/negativity for lupus anticoagulant as defined by the authors

Participant-level information: maternal outcomes after trial entry:

1) Allocated intervention group (for example, IvIg, placebo, no intervention)

2) Time of first infusion (for example, before pregnancy or gestational week in pregnancy)

3) Dose and number of infusions

4) Pregnancy outcome (for example, miscarriage, live birth, ectopic pregnancy, induced abortion, stillbirth)

5) Time of pregnancy loss/live birth (gestational age)

6) SAEs

7) AEs

8) Quality of life

Participant-level information: infant outcomes:

1) Gestational age at birth

2) Sex

3) Birth weight

4) SAEs

5) AEs

6) Quality of life

### Risk of bias (quality) assessment

We will use the instructions in the *Cochrane Handbook for Systematic Reviews of Interventions*[25] and The Cochrane Hepato-Biliary Group Module [26] in our evaluation of the methodology and hence bias risk of the included trials [27-32]. Again, two review authors (PE and JL) will assess the included trials independently of each other. We will evaluate the methodology in respect of generation of allocation sequence, allocation concealment, blinding of participants and treatment providers, blinding of outcome assessors, incomplete outcome data, selective outcome reporting, for-profit bias, and other bias sources. This is done because these components enable classification of randomised trials with low risk of bias and high risk of bias. The latter trials overestimate positive intervention effects and underestimate negative effects.

We will classify the trials according to the components below:

#### Generation of allocation sequence

‘Low risk of bias’: if the allocation sequence is generated using a computer or a ‘random number table’.

‘Uncertain’: if the procedure in respect of randomisation is not well described.

‘High risk of bias’: if the trial uses, for example, alternation for allocating of participants.

#### Allocation concealment

‘Low risk of bias’: if the allocation sequence is concealed from the investigators, treatment providers and participants, for example by central randomisation, and this procedure is described and documented.

‘Uncertain’: if the procedure to conceal allocation is not sufficiently described.

‘High risk of bias’: if the investigators, treatment providers and the participants are able to predict the allocation sequence.

### Blinding of the participants and treatment providers

‘Low risk of bias’: if the participants and the treatment providers are blinded to treatment allocation and this is described. The placebo infusions should be identical to the immunoglobulin infusions regarding appearance, colour and solubility.

‘Uncertain’: if the procedure of blinding is insufficiently described.

‘High risk of bias’: if blinding is not performed or the trial uses ‘no intervention’ as control intervention.

#### Blinding of outcome assessors

‘Low risk of bias’: if the trial investigators performing the outcome assessments are blinded to the treatment allocation and this is described.

‘Uncertain’: if the procedure of blinding is insufficiently described.

‘High risk of bias’: if blinding is not performed.

#### Incomplete outcome data

‘Low risk of bias’: if dropouts following randomisation can be described as being similar in the two intervention groups, and if the trial allows intention-to-treat analysis using proper methodology, for example, multiple imputations.

‘Uncertain’: if dropouts are not stated, or if the reasons why the participants dropped out are unclear.

‘High risk of bias’: if the pattern of dropouts can be described as being different in the two intervention groups.

#### Selective outcome reporting

‘Low risk of bias’: if all outcomes are stated in the results, and the hierarchy of the outcomes are documented in a protocol before launch of randomisation.

‘Uncertain’: if the method of choosing outcomes is inadequately described.

‘High risk of bias’: if there is incongruence between the original protocol and the outcome measures used in the results, or if not all of the outcome measures are stated.

#### For-profit bias

‘Low risk of bias’: if the trial is not financed by a company that might have an interest in a given result.

‘Uncertain’: if there is no description of how the trial is financed.

‘High risk of bias’: if the trial is financed by a company that might have an interest in a given result.

#### Other sources of bias

If other sources of bias are evident these sources of bias will be presented and the implications will be discussed and considered in the assessment of treatment effects.

#### Overall assessment of risk of bias

A trial will be classified as ‘low risk of bias’ only if all of the bias components described in the above paragraphs are classified as ‘low risk of bias’. If one or more of the bias components are classified as ‘uncertain’ or ‘high risk of bias’ the trial will be classified as ‘high risk of bias’.

In case that we find no trials with low risk of bias or only find very few trials with low risk of bias, we plan to identify a group of trials with ‘lower risk of bias’ defined as those having low risk of bias in the domains: generation of allocation sequence, allocation concealment, and blinding of the participants and treatment providers.

#### Assessment of reporting bias

Different types of reporting biases (for example, publication bias, time lag bias, outcome reporting bias, and so on) will be handled following the recommendations of the *Cochrane Handbook for Systematic Reviews of Interventions*[25]. On all outcomes, we will test for funnel plot asymmetry when there are at least ten trials included in the meta-analysis. For continuous outcomes with intervention effects measured as mean difference, the test proposed by Egger et al. will be used for test for funnel plot asymmetry [33]. We will take into account that asymmetric funnel plots are not necessarily caused by population bias, and publication bias does not necessarily cause asymmetry in a funnel plot.

#### Individual patient data (IPD)

By using IPD we avoid the potential biases of the published aggregate data (AD) as we can use consistent inclusion/exclusion criteria across the included randomised clinical trials. One potential problem for IPD analyses is that IPD may not be available from all the trials and results from an IPD-only meta-analysis may be biased if unavailability of IPD is related to the trial results. To avoid the bias we will supplement the available IPD with AD for those studies where IPD are not available.

### Strategy for data synthesis

We will undertake meta-analyses according to the recommendations stated in the *Cochrane Handbook for Systematic Reviews of Interventions*[25]. For binary outcomes we will calculate a standard estimation of the risk ratio (RR) and its 95% confidence interval (CI). For continuous outcomes we will estimate the mean difference (MD) between groups. We prefer not to calculate effect size measures (standardized mean difference, SMD). However, if scales of very considerable similarity are used, we can presume there is a small difference in the different measurements, we will calculate effect size and transform the effect back to the units of one or more of the specific instruments. All meta-analyses will be performed both with a fixed-effect and a random-effects model.

We will also perform trial sequential analyses on the outcomes, in order to control the risks of type I and type II errors that occur in traditional meta-analyses due to sparse data and repetitive analyses of accumulating [34-39]. In order to control these risks we will calculate the diversity-adjusted required information size and assess the eventual breach of the cumulative Z-curve of the relevant trial sequential monitoring boundaries. A more detailed description of trial sequential analysis can be found at http://www.ctu.dk/tsa/. For binary outcomes we will estimate the diversity adjusted required information size based on the proportion of patients with an outcome in the control group, a risk ratio of 20% or as suggested by the trials with low risk of bias, an α of 5%, a β of 20%, and diversity of 30% and 60%, or as suggested by the trials in the meta-analysis. For continuous outcomes we will estimate the diversity-adjusted required information size based on the standard deviation (SD) observed in the control group of trials with low risk of bias and a minimal relevant difference of 50% of this standard deviation, an α of 5%, a β of 20%, and diversity of 30% and 60%, or as suggested by the trials in the meta-analysis.

For all outcomes and according to availability we will combine IPD and published AD into a pooled effect measure using Riley’s two stage method [40]. In the two-stage method the available IPD are first reduced to AD in each trial and these AD (from the IPD studies) are combined with the existing AD (from the AD trials) using standard meta-analyses AD techniques. All meta-analyses will be performed both with a fixed-effect and a random-effects model.

### Dealing with missing data

#### Dichotomous data

For dichotomous outcomes, we will analyse data according to the intention-to-treat principles, whereby all participants randomised in each trial are included in the analyses. Participants with missing outcome data will initially be considered as having: 1) no live birth; 2) no SAEs (for the women); and 3) no AEs (for the women).

For women with live birth, we will, in the primary analyses, impute missing values assuming that the participants missing at follow-up had: 1) no SAEs (for the babies); 2) no premature birth; 3) equal number of live born boys and girls; 4) no low birth weight; and 5) no AEs (for the babies).

#### Continuous data

If SDs are not reported they will be calculated if this is possible using other data from the trial. If calculation is impossible, the SDs will be imputed from trials with similar characteristics.

### Sensitivity analyses

#### Assumption for lost dichotomous data

We will perform three sensitivity analyses:

1) ‘Best-worst-case’ scenario: It will be assumed that all women lost to follow-up in the experimental group had no outcome and all women lost to follow-up in the control group had the outcome.

2) ‘Worst-best-case’ scenario: It will be assumed that all women lost to follow-up in the experimental group had the outcome and all women lost to follow-up in the control group had no outcome.

3) Per-protocol analysis: We will analyse the outcomes after exclusion of women with 1) ectopic pregnancies; 2) miscarriages with a chromosome abnormal foetus; 3) genetic termination and 4) women, who did not complete the intervention as defined by the authors.

#### Assumptions for lost continuous data

Where assumptions have to be made regarding missing SDs (see: Dealing with missing data), sensitivity analysis will be undertaken testing how prone results are to change when 'completer' data only are compared to the imputed data using the above assumption. If there is a substantial difference, we will report results and discuss them but continue to employ our assumption.

### Subgroup analyses

If the necessary data are available, the following subgroup analyses will be conducted:

1) Trials with ‘low risk of bias’ compared to trials with ‘high risk of bias’, or if we find no trials with ‘low risk of bias’, we will compare trials with ‘lower risk of bias’ to trials with ‘high risk of bias’

2) Participants with ≥ three miscarriages compared to participants with fewer miscarriages

3) Participants with ≥ four miscarriages compared to participants with fewer miscarriages

4) Participants with secondary RM compared to participants with primary RM

5) Trials providing doses of IvIg ≥ the median dose in grams in all trials from before conception to gestational week 10 in all trials compared to trials providing IvIg doses < median

6) Patients without lupus anticoagulant and/or IgG anticardiolipin as defined by the authors compared to patients with lupus anticoagulant and/or IgG anticardiolipin as defined by the authors

## Discussion

A great mental strain is imposed on a woman if she is not being able to give live birth. Thus, a miscarriage results in great sorrow, loss of life quality, and personal concern. In particular, RM is extremely stressful [2]. It is, therefore, very important to conduct research in this area. For women with RM, there is currently no scientifically evidence-based treatment, which significant could improve their ability to give live birth. In a Cochrane systematic review on treatment of women with RM with antiphospholipid antibodies or lupus anticoagulant, it was concluded that although the combined treatment with heparin and low dose aspirin may reduce miscarriage rate in the patients, the quality of the trials was high due to lack of allocation concealment; furthermore, the participant characteristics varied very much between the trials [41].

At least eight randomised placebo-controlled trials investigating IvIg with a total of 324 RM patients have been published, with conflicting results [12,17-23]. These differences could in theory be explained by the fact that the trials conducted until now have been very heterogeneous with regard to the selection of patients, doses of IvIg provided and starting time of infusions. The latest systematic Cochrane review on the topic was conducted in 2006 and included data from seven placebo-controlled trials [11]. The review concluded that there was no significant treatment effect. However it should be noted that no subgroup analyses were implemented. The treatment is very expensive and could imply potential SAEs. It is, therefore, very important to detect the relevant patient group for this treatment if it exists.

We will, in our systematic review, include data from the newest randomised placebo-controlled trial, which we expect will be published in 2014. We will also include IPD in our analyses. This would make it possible to carry out important subgroup analyses with the aim of evaluating in which subgroup the treatment might have the highest impact. There is according to our knowledge no published systematic review on the topic which has used IPD in the analyses.

## Abbreviations

AD: aggregated data; AE: adverse event; ß-hCG: beta-human chorionic gonadotropin; CI: confidence interval; IPD: individual patient data; IvIg: intravenous immunoglobulins; RM: recurrent miscarriages; RR: risk ratio; SAE: serious adverse event; SD: standard deviation; SMD: standardised mean difference; TSA: trial sequential analyss.

## Competing interests

One of the review team members, professor Ole B Christiansen (OBC), is investigator in three of the randomised clinical trials in the topic. To avoid potential bias, OBC will not proceed in the selecting of relevant trials or in the data analyses. This will be done by the remaining review team members.

The remaining review team members (PE, JL, CG) declare that they have no competing interest.

## Authors´ contributions

PE participated in the development of the design of the protocol and drafted, edited and finalised the manuscript. JL participated in the development of the design of the protocol and revised the manuscript critically. CG participated in the development of the design of the protocol and revised the manuscript critically. OBC initiated the idea for the systematic review, participated in the development of the design of the protocol and revised the manuscript critically. All authors read and approved the final manuscript.
